# Osmolyte Induced Tumorigenesis and Metastasis: Interactions With Intrinsically Disordered Proteins

**DOI:** 10.3389/fonc.2018.00353

**Published:** 2018-08-28

**Authors:** Franklin D. Rumjanek

**Affiliations:** Instituto de Bioquímica Médica Leopoldo de Meis, Universidade Federal do Rio de Janeiro, Rio de Janeiro, Brazil

**Keywords:** metastasis, intrinsically disordered proteins, transcription factors, osmolytes, conformation

## Abstract

In spite of a great deal of work, the biochemical mechanisms underlying tumorigenesis and metastasis are not yet fully understood. Specifically regarding metastasis many authors consider that malignancy is caused by the accumulation of mutations. However, evidence is gathering to show that tumors are composed of heterogeneous cell populations subjected to selective pressures. In this micro evolutionary scenario, intra- and extra-cellular selective pressures will determine which subpopulations of tumor cells will thrive and be able to dissociate from the tumor as autonomous metastatic cells. We propose here that alteration of conformations of transcription factors confer novel non-canonical functions that may induce oncogenesis and metastasis in a mutation independent manner. We argue that the functional plasticity of transcription factors is due to intrinsically disordered domains (IDRs) of proteins. IDRs prevent spontaneous folding of proteins into well-defined three-dimensional structures. Because most transcription factors contain IDRs, each could potentially interact with many ligands. This high degree of functional pleiotropy would then be ultimately responsible for the metastatic phenotype. The conformations of proteins can be altered by chemical chaperones collectively known as osmolytes. Osmolytes are small organic molecules permeable through biological membranes that can accumulate in cells, increase the thermodynamic stability of proteins, modulate enzyme activity and prevent protein aggregation. Thus, by modifying IDRs, osmolytes could subvert the homeostatic regulatory network of cells. Untargeted metabolomic analysis of oral cancer cells showed that those with the greatest metastatic potential contained several osmolytes that were absent in the non-metastatic cells. We hypothesize that high concentrations of osmolytes might promote conformational alterations of transcription factors that favor metastatic behavior. This hypothesis is eminently testable by investigating whether: (a) the intracellular microenvironment of metastatic cells differs from non-metastatic cells and whether osmolytes are responsible for this change and (b) high intracellular concentrations of osmolytes are sufficient to induce structural modifications in regulatory protein so as to establish novel interactive networks that will constitute the metastatic phenotype. Synthetic cell penetrating peptides mimicking IDRs could act as sensitive probes. By exposing the peptides to the microenvironments of living tumor and metastatic tumor cells one should be able to compare the chemical shifts as revealed by spectra obtained by nuclear magnetic resonance (NMR).

## Background

Metastasis is responsible for about 90% of cancer deaths and still represents the greatest medical challenge regarding control of this chronic disease. Interestingly, the vast majority of metastatic tumors are epithelial in origin. The fundamental processes occurring during the embryonic development of tissues and to a large extent also during wound healing depends largely on the so-called epithelial-mesenchymal transition (EMT). The EMT consists of a sequence of events that regulate the dynamic association-dissociation of epithelial cells, which reflect many of the morphogenetic stages that occur during embryogenesis. In pathological situations such as metastasis, cells that dissociate from the primary tumor retrieve some of these embryogenic changes, that is, the dissociated cells acquire characteristics similar to those of undifferentiated mesenchymal cells whose morphology and properties are characteristically distinct from epithelial cells. Therefore, it is not surprising that the deregulation of the signaling pathways that take part in EMT can generate self-sufficient mesenchymal cells that may eventually behave as metastatic cells. For the purposes of the hypothesis described here, tumorigenesis and metastasis will be considered as occurrences that to a certain extent depend on intrinsic factors already present in the tumor cells and largely on extrinsic factors supplied by the microenvironment. With regards to the metastatic phenotype of tumor cells many questions remain unanswered. For example, why are some tumors highly likely to form metastases while others do so rarely? Why do some tumor types exhibit tropisms for certain tissues? What are the mechanisms underlying the resistance to anoikis? In what way do the metastatic cells survive the stress generated during extravasation when crossing the endothelium of blood and/or lymphatic vessels and subsequent migration toward the target tissues? What mechanisms are used by metastatic cells to invade target tissues? From which metabolic pathways do tumor cells harness the energy to perform and sustain all processes linked to proliferation and metastasis? Traditionally, all regulatory genes that control the major cellular events associated with EMT, proliferation and resistance to anoikis are considered to arise from driver mutations that produce either gain or loss of function that could induce super expression or repression of key transcription factors acting as oncogenes and tumor suppressors. The net result of the gain or loss of function is the expression of traits that endow the cells with a non-social behavior such as immortality and invasiveness typical of malignant tumors. However, oncogenesis and metastasis need not derive from the progressive accumulation of driver mutations. A fundamental premise put forward here is that potentially metastatic cells already contain within themselves the features, morphogenetic or not, that will determine which phenotypes will prevail upon selection. In other words, a tumor may harbor subpopulations of cells that are already programed to dissociate, migrate and invade other tissues. This view (born to be bad) has been supported by a recent report in which mutational patterns have been correlated to early colorectal tumor cell mobility (1). This is in keeping with the notion that tumors that become malignant, do so mainly by the accumulation of mutations as proposed by Fox et al. (2). Mutations are also the basis of the work of Tomasetti and Vogelstein (3, 4) who developed the “bad luck” hypothesis. According to this proposal the etiology of cancer correlates with the number of stem cell divisions of various tissues. This model relies on driver-type mutations present in oncogenic stem cells. However, mutations cannot be the sole explanation. One has to take into account the tumorigenic effect of the environment, including the contribution of stromal cells. Indeed, demographic studies on a global scale have shown quite clearly that the incidence of various types of cancer follows a heterogeneous geographical distribution. Furthermore, individuals moving from one region to another acquire the risk determined by that region. Accordingly, Little et al. (5) argue that the effect of the environment is actually stronger than that due to mutations. This assertion makes sense from an evolutionary point of view; the microenvironment does exert tiered selective pressures on tumor cells, including those imposed by drugs in the case of individuals undergoing chemotherapy. Thus, the various stages of metastasis–EMT, dissociation of the original tissue cells, intravasation into the lumen of blood or lymphatic vessels, migration along the circulation, organ attachment distant from the primary tumor, extravasation, and colonization–are seemingly general manifestations that may be regarded as modifications selected from the pool of subverted pathways underlying tumorigenesis. What mechanisms could drive the establishment of metastatic phenotypes? By following the evolutionary approach, rather than exclusively invoking the fixation of random driver mutations, one should allow for the existence of intracellular sensors that respond to stress by generating new cellular functions. These may emerge as a result of the recruitment of pre-existing pathways originally associated with different physiological roles in the cell, rather than to assume that cells “progress” from normal to tumoral, or from tumoral to metastatic due to the accumulation of mutations. This recruitment phenomenon or gene shuffling lies at the heart of exaptation, a term coined by Stephen Jay Gould (6), which by replacing adaptation avoids the teleological undertones contained therein. The exaptation model as applied to tumor cells predicts that, in the case of tumor and metastatic tumor cells, proteins with well-established functions may in fact, depending on changes affecting the intracellular microenvironment, acquire novel roles. As a matter of fact, recent literature shows that many proteins can be classified as the so called “moonlighting proteins” (7–9). Within this framework it is important to consider that the acquisition of new functions by the moonlighting proteins may not necessarily derive from modifications in their primary structures (gene duplication, alternative splicing), or in any way dependent on point mutations at the gene level. Functional plasticity can also be generated by post-translational modifications, such as acetylation and protein phosphorylation (10). Other situations compatible with exaptation may be relevant to metastasis such as alterations in the redox equilibrium (11), collective migration of tumor cells (12) and the effect of monocarboxylate transporter 1 (MCT1) on the switch from lactate-fueled respiration to glycolysis (13). The above examples illustrate the potential of establishing non-canonical interactions in both, metabolic and signaling pathways. Thus, exaptation can introduce novelty by way of increasing plasticity in cellular physiology. This could be beneficial (evolutionary), or deleterious as in the case of cancer and other chronic pathologies. In addition to the post-translational modifications mentioned above it is possible to resort to allostery as mutation independent factors that could contribute to the increase of plasticity. For example, it is known that in cell culture conditions, the composition of the media (glucose, glutamine, and other metabolites) promptly influence the activity, and in the long range, the expression profile of enzymes involved in intermediary metabolism (14). These observations highlight the fact that cells are immediately and transiently responsive to changes in the cell's microenvironment. Although it is known that these allosteric adjustments are part of the normal homeostatic mechanisms, for the sake of the hypothesis developed here it is important to keep in mind that small organic molecules such as metabolites do affect protein conformation and thus could be considered as activity modulators. Also, it is important to bear in mind that the plasticity mediated by small organic compounds surrounding proteins although reversible when acting as part of the normal homeostatic control, could become permanent under special conditions. This is the case of the transition of certain prion-like proteins between soluble and amyloid-like states (15). Even more strikingly, it has been reported that some traits acquired through self templating conformations of prion-like proteins were heritable, thus reinforcing the occurrence of the so called protein-based inheritance (16). Curiously and suggestively, the same group was able to show that many proteins that underwent self-templating conformations were transcription factors and RNA binding proteins. This dynamic interplay may then be more relevant to pathogenesis than has been previously thought. Collectively those results strengthen the idea that the changes in the microenvironment are in their own right sufficient to induce profound and long lasting effects on cell physiology mediated by modified effectors that can actually be passed on along several generations. Inflammation may illustrate this situation. Although reversible in the acute stage, chronic inflammation, which in many types of cancer is consensually regarded as a pre-tumoral state (17), could reflect a situation of “fixed” traits propagated by protein-based inheritance. The contention here is that certain conformational states acquired by regulatory proteins may exhibit high adaptive values and thus may be partly responsible for tumorigenesis and metastasis. The possibility that allosteric alterations may be involved would add new layers of complexity to the regulatory systems. Thus, the occurrence of mutations as the sole or main causative agents of cancer may have to be reviewed under the light of exaptation especially by considering that the majority of mutations are of the passenger type (18).

What are the preferential sites producing conformational alterations in proteins? Structural analysis of proteins has established that the majority contain domains that fold on to form well-defined three-dimensional structures, such as alpha helices and beta conformations (pleated beta sheets). Others, however–about 1/3–display domains that lack these ordered structures. These domains are known as intrinsically disordered domains (IDRs) (19). In order to be classified as an IDR a domain requires a particular primary structure. For example, these domains appear to be essentially polar, displaying relatively long stretches of amino acids such as glutamic acid (at neutral pH these domains will not form alpha-helices). Additionally domains that prevent folding usually contain few amino acids with hydrophobic side chains, especially aromatic rings. IDRs may exhibit net charge and be enriched in proline (this prevents the formation of hydrogen bonds and thus introduce instability in the alpha-helix). The net result is the formation of localized disordered structures that afford proteins with greater conformational flexibility. Proteins containing extensive IDRs are called intrinsically disordered proteins, IDPs (20). Because of this flexibility, disordered regions are highly dynamic with respect to their interactions with various ligands. Due to the resulting low binding affinity, the bonds formed are transient and exhibit diminished specificity. In other words, IDPs are quite promiscuous as far as their ligands are concerned. Interestingly, the proteins that are functionally classified as transcription factors are the very ones that commonly display IDRs (21). Transcription factors bind to both DNA in the promoter region and to co-activators of the transcription and remodeling machinery of various chromatin elements. Classic examples are bZIP (Basic Region Leucine Zippers) transcription factors that play important roles in the regulation of eukaryotic genes (22). Such structural features were also found in mitochondrial proteins, ribosomal proteins and nuclear proteins (21, 23). Therefore, it is evident that in addition to being abundant in nature, IDPs occupy central positions in virtually all processes associated with the fine regulation of cellular physiology, including those whose deregulation may lead to oncogenesis, namely the cell cycle, proliferation and differentiation (24). It is important to realize that the binding-induced annealing created by those transcription factors bearing IDRs potentially create new connections that may bear on oncogenesis and metastasis. Because of the functional diversity promoted by the IDRs there is a growing awareness that the promiscuity exhibited by IDPs may be the underlying mechanism of the so called “moonlighting proteins.” These are proteins that historically were recognized as having only one function, but that later were found to participate in other pathways, particularly those involved in regulation. For example, enzymes of the intermediary metabolism (8, 9). In the scenario of IDPs and signaling pathways it is possible to understand why certain proteins such as p53, HIF-1A, c-Myc, K-Ras and many others are classified as “hub” proteins. In reality these proteins are only central to several signaling pathways precisely because their structures contain a large proportion of IDRs (25–27). Taking into account the above considerations about IDPs it is reasonable to propose that the phenotypes of the tumor and metastatic cells may arise as a result of a functional reprogramming dictated by the relatively loose interactions mediated by IDR containing proteins. What factors can change the conformations of IDRs and IDPs in physiological situations? In cells, nascent proteins are gradually folded in the endoplasmic reticulum sometimes aided by molecular chaperones, a family of proteins (hsp70 and hsp90, for example) that not only direct the correct folding of the peptides, but also prevent the formation of aggregates that otherwise could accumulate and insolubilize within the cells (28). The role of heat shock proteins in cancer has been well established (29). However, in addition to the accessory role of molecular chaperones, the three-dimensional structures of mature proteins are also affected by osmolytes, also known as chemical or organic chaperones. Osmolytes are small organic molecules that are divided into three main groups: amino acids or derivatives thereof (glycine and taurine, for example), polyhydroxylated compounds such as glycerol, inositol and sorbitol and amine oxides such as n-trimethylamine oxide and choline sulfate. Historically, these natural compounds were named osmolytes because they were associated to the preservation of osmotic equilibrium of cells under situations of stress-thermal, oxidative, osmotic or accumulation of urea. In these stress situations the osmolytes accumulate inside the cells and can reach relatively high concentrations (up to about 400 mM in some cases). The increase in the local concentration of the osmolytes may occur by *de novo* synthesis, and/or by increased activity of specific transporters. High intracellular concentrations of protective or stabilizing osmolytes increase the thermodynamic stability of the proteins without affecting other cellular processes, i.e., even in high concentrations osmolytes are not cytotoxic (30, 31). Under non-denaturing conditions the interaction between the osmolytes and the peptide backbone is unfavorable, a process called the osmophobic effect. Conversely, denaturing osmolytes tend to accumulate on the surfaces of proteins. Thus, the protein folding equilibrium will depend ultimately on the balance between the opposing forces of the osmophobic effect and denaturation (32). Regeneration of the native conformation through the osmophobic effect can be equated to an increase in the free energy of proteins relative to the denatured state. In other words, those protective osmolytes that bind to a protein reduce the ability of water molecules to solvate it and therefore denaturation is unfavorable, that is, the osmolytes render the proteins more thermodynamically stable than they would be in the presence of water (33). The above considerations raise the question of what would be the effect of the osmolytes on IDRs and IDPs. How would the IDR peptide backbone react to osmolytes? Would the IDRs exhibit the osmophobic effect? Recent results show that the osmolyte trimethylamine *N*-oxide (TMAO) was able to preferentially modify the conformation of IDPs and abolish their functions (34). Likewise, trehalose, a non-reducing disaccharide osmolyte, has been shown to promote the transition from the intrinsically disordered α-synuclein protein to ordered, i.e., directly contributed to the generation of a non-native conformation (35). Whilst data on the various *de facto* properties of IDR containing proteins exist in many different contexts, proof that osmolytes can actually be tumorigenic is still lacking. Nevertheless, the arguments presented here make a case for this class of organic compounds as modulators of key components of signaling pathways. By extension, this hypothesis also underlines the importance of the intracellular physicochemical microenvironment in pathogenesis in a mutation independent manner.

### Osmolytes and tumor cells

**Figure 1:**
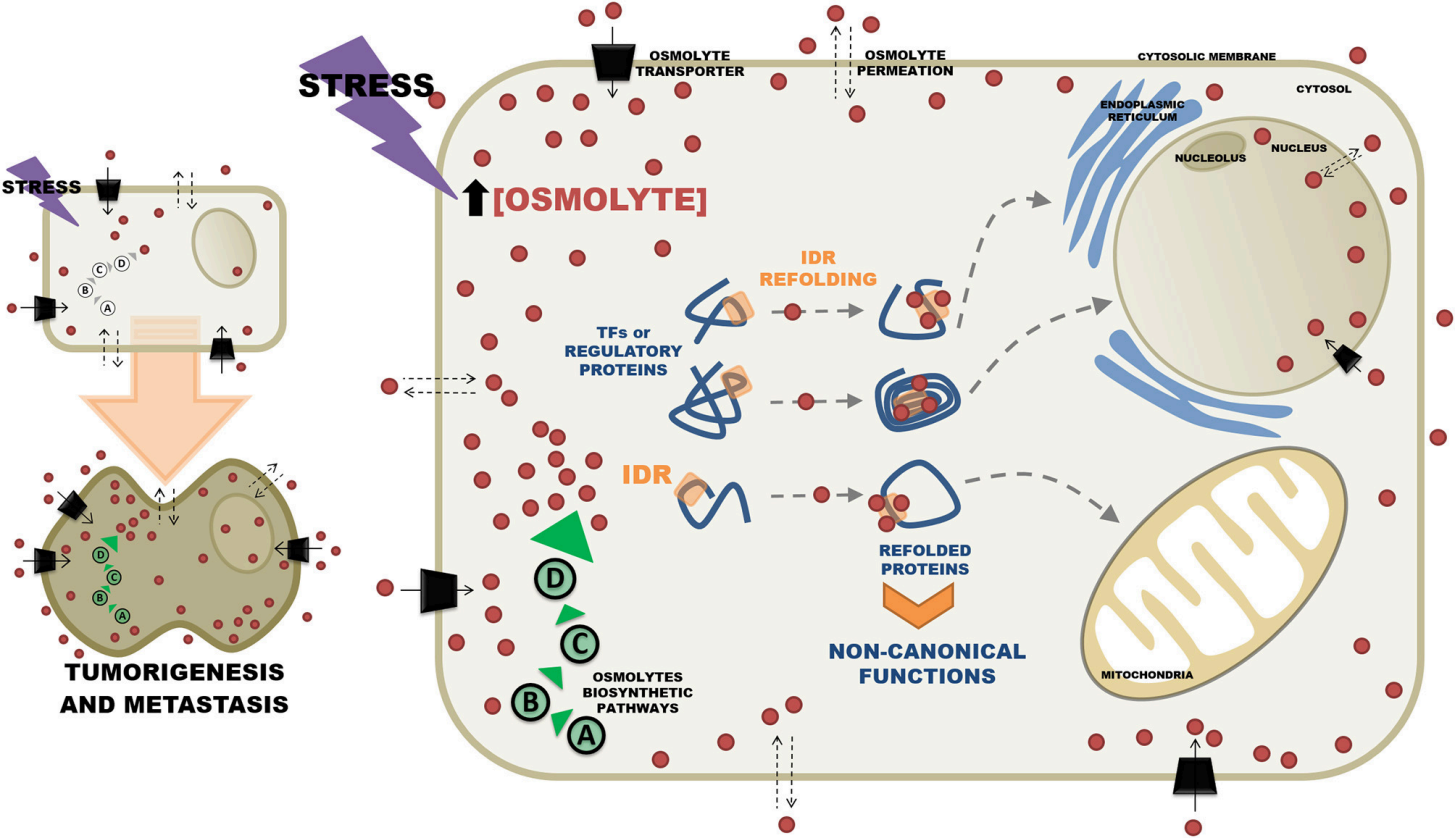
Effects of osmolytes on the 3D conformations of intrinsically disordered proteins. In stress situations osmolytes (light brown small circles) can be either synthesized intracellularly by different pathways (**A–D**, encircled in green), or can permeate the cytosolic membrane from the extracellular space. Alternatively osmolytes can be incorporated by the cells via specific transporters (black parallelograms). Increased intracellular concentrations of osmolytes induce refolding of intrinsically disordered regions (IDR) of transcription factors (TF) and other regulatory proteins. In the refolded state proteins acquire novel properties that mediate non-canonical interactions with individual members of signaling pathways. Refolding of TFs can also bring together aminoacids that will form sub cellular localization signals that will atypically direct them to various subcellular organelles. Sustained stress conditions may contribute to chronic osmolyte imbalance and eventually lead to tumorigenesis and metastasis.

Data published by our group have already shown that metastatic cells display a metabolic profile different from those of non-metastatic tumor cells, or normal cells, especially with respect to oxidative metabolism (36). Interestingly, additional unpublished results employing microcalorimetry have shown that in several tumor cell lines, metastatic cells are the ones that consistently release more heat, suggesting that they have either a differentiated energetic metabolism and/or that in metastatic cells protein-protein interactions are more extensive, reflecting a differentiated interactome. In this case, the mechanism underlying the differentiated interactome could have its origin in the conformational changes putatively promoted by the osmolytes. Consistently, in a recently published paper, we have shown that the metabolome of metastatic cells displays qualitative and quantitative differences with respect to non-metastatic cells. Furthermore, we showed that a good proportion of the differences found among metabolites could be assigned to several osmolytes such as threitol and amino acids (37). These results reinforce the idea that in addition to a distinct metabolic reprogramming, the metabolites detected by NMR may reflect the existence of an altered intracellular microenvironment in the metastatic cells. In this context, the recent observation that the metabolomic analysis of prostate cancer highlighted the presence of inositol (an osmolyte) as the main predictor of aggression in these tumors is very suggestive (38). Thus, in addition to the already well described roles of inositol as the second messenger of hormones acting on the regulation of cytosolic Ca2^+^ and as regulator of the oncogenic pathway PI3K (39), this compound could also be acting as an osmolyte thus contributing to modifications of the conformations of proteins, especially those functionally classified as transcription factors. Therefore, considering the scenario described above, it is relevant to ask the question whether the changeable intracellular microenvironment of tumor cells can promote the formation of novel signaling pathways that would drive cells toward metastasis. The signaling pathways would be composed of regulatory proteins containing intrinsically disordered domains and the osmolytes would act as effectors promoting alternative protein folding. Figure 1 illustrates this hypothesis. In Figure 1, the stress induced intracellular osmolyte concentrations increase either by permeation through the membranes or by enhancement of intracellular osmolyte biosynthetic pathways. High intracellular concentrations of osmolytes change the conformation of IDR containing transcription factors thereby altering their binding properties and consequently their function. Furthermore, refolded proteins could display new trafficking properties through the acquisition of sub cellular localization signals that would allow them to perform different functions in various organelles. In addition to effects on the structures of transcription factors and regulatory proteins, osmolytes might also affect the structures of DNA and RNA and thus directly contribute to modulation of cellular function by altering nucleic acid conformational transitions (40, 41).

Experimental confirmation of this hypothesis would require the means to prospect alterations in the intracellular microenvironment of living tumor cells displaying different degrees of metastatic potential. This could be achieved by probing living cells with peptides bearing IDRs and measuring the ability of the intracellular milieu to differentially modify their conformations. In addition to bearing aminoacids labeled with ^13^C and ^15^N, the primary structure of the synthetic peptides would also contain sequences found in cell penetrating peptides (42) to allow their incorporation by cells. Changes in the secondary structure of sensor peptides induced by osmolytes could be monitored in living cells by non-invasive methods such as nuclear magnetic resonance (NMR). With this experimental approach it would be possible to comparatively detect the chemical shifts of the sensor peptides. In order to investigate whether osmolytes are in fact responsible for the conformational changes of IDR containing peptides a series of *in vitro* experiments could be conducted attempting to reproduce the conformational changes (chemical shifts) observed intracellularly. For these experiments sensor peptides would be added to solutions containing individual osmolytes or mixtures of these compounds. The *in vitro* experiments with living cells could potentially evidence osmolyte induced intracellular modifications of protein conformation. Demonstrations of the *in vivo* effect of osmolytes as inducers of oncogenesis and metastasis may resort to experiments using, for example, mouse xenograft models, in which the explanted cells had been transfected with constructs mediating the overexpression of enzymes participating in the biosynthesis of osmolytes. These experiments could detect tumor growth and invasiveness.

## Conclusions

We propose that the intracellular microenvironment of tumor cells is able to generate the metastatic phenotype by inducing conformational changes of transcription factors and that osmolytes could be the effectors responsible for those modifications. Furthermore, the conformational changes could be transferred from one generation of cells to another following a prion-like model of protein based inheritance. The functional plasticity of transcriptional factors has been amply documented in the literature and places them as hub proteins in several signaling pathways. This model is based on the observation that the functional plasticity of the transcription factor proteins can be associated to the occurrence of intrinsically disordered regions in their structures. These IDRs allow transcription factors to interact with many different ligands and hence would explain the emergence of traits associated with metastasis. Based on published experimental results we identify osmolytes as the potential effectors promoting these structural alterations and hypothesize that the alterations in the metabolic pathways producing osmolytes or their uptake may contribute to the generation of the metastatic behavior of tumor cells. It is expected that the NMR spectra of the intracellular sensor peptides will be able to confirm not only that the intracellular milieu of metastatic cells differs from that of non-metastatic cells, but also that osmolytes can induce the conformational changes.

## Author contributions

The author confirms being the sole contributor of this work and approved it for publication.

### Conflict of interest statement

The author declares that the research was conducted in the absence of any commercial or financial relationships that could be construed as a potential conflict of interest.
